# Inverted ductal papilloma: report of a rare case and review of the literature

**DOI:** 10.4317/jced.62199

**Published:** 2024-11-01

**Authors:** Carla Isabelly Rodrigues-Fernandes, Anne Evelyn Oliveira Moura, Danielle Machado Farias, Elaine Judite de Amorim Carvalho, Pablo Agustin Vargas, Danyel Elias da Cruz Perez

**Affiliations:** 1 DDS, PhD. Oral Pathology Unit, Department of Clinic and Preventive Dentistry, Universidade Federal de Pernambuco, Recife, Pernambuco, Brazil; 2 DDS. Department of Oral Diagnosis, Semiology and Pathology Areas, Piracicaba Dental School, University of Campinas, Piracicaba, São Paulo, Brazil; 3 DDS, MSc. Oral Pathology Unit, Department of Clinic and Preventive Dentistry, Universidade Federal de Pernambuco, Recife, Pernambuco, Brazil; 4 DDS, PhD. Department of Oral Diagnosis, Semiology and Pathology Areas, Piracicaba Dental School, University of Campinas, Piracicaba, São Paulo, Brazil

## Abstract

Inverted ductal papilloma is an uncommon benign papillary endophytic tumor. This report aimed to present a case of inverted ductal papilloma of the oral cavity. A 54-year-old female patient presented with an asymptomatic nodular lesion on the lower lip. Time of duration was undetermined. The diagnostic hypotheses were fibrous hyperplasia and benign mesenchymal neoplasm. An excisional biopsy was performed, and microscopic examination showed an endophytic proliferation of squamous cells with the presence of microcysts, and goblet cells arranged in nest and islands. No cellular atypia was observed. The neoplastic cells were positive for CK7, and CK14. Proliferation index, demonstrated by Ki-67, was positively restricted to the basal tumor layer. The final diagnosis was oral inverted ductal papilloma. No signs of recurrence were found after surgery.

** Key words:**Inverted papilloma, intraductal papilloma, oral papilloma.

## Introduction

Benign papillary lesions arising from the main excretory salivary ducts include intraductal papilloma and inverted ductal papilloma. This group comprise about 0.3% of oral salivary gland tumors (1). Inverted ductal papilloma was first described in 1982 (2). The name was proposed because of the similarity to the papilloma of the urinary bladder, nasal cavity, and paranasal sinuses (3,4). Most involved sites are intraoral minor salivary glands (1,5).

The etiology of oral inverted ductal papilloma (OIDP) remains undetermined (6). Preferential sites of OIDP are diverse and include the buccal mucosa, lower lip, vestibular area, soft palate, and floor of the mouth (1,2,7,8).

Oral inverted ductal papilloma (OIDP) is a rare endophytic tumor (6). Forty cases of OIDP have been published in the English-language literature so far (2,5,9). Hence, we report a case of OIDP in a middle-aged woman, emphasizing the clinical, histopathological and immunohistochemical findings, and provide a literature review on this topic.

## Case Report

**Figure 1:**
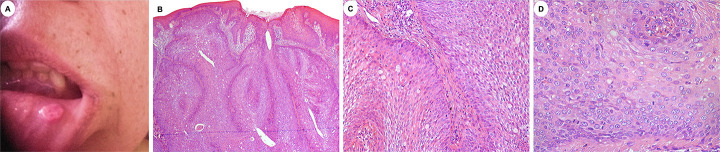
Clinical and microscopic features of OIDP. A: A round-shaped, nodular lesion located in the left labial mucosa. B: Papillary endophytic proliferation with preponderance of squamous cells with clear cells and microcystic spaces. Cleft-like structures are also noted. The lesion is covered by a mature squamous cell epithelium (H&E, 50x). C: Islands of neoplastic cells alongside with goblet cells in a fibrous stroma with mild inflammatory infiltrate (H&E, 200x). D: Higher power view of the tumor exhibiting epidermoid cells with moderate cytoplasm and vesicular nuclei. Few typical mitotic figures are also observed (H&E, 400x).

A 54-year-old female patient presented with an asymptomatic lesion on the lower lip, with unknown time of duration. The patient did not present any relevant health problems. Extraoral examination did not reveal any asymmetries or adenopathy. Intraoral examination exhibited a well-defined, round-shaped nodular lesion, with a smooth, slightly erythematous, and non-ulcerated surface, located in the left labial mucosa (Fig. 1A). It measured approximately 1.0 cm and exhibited tender consistency. There were no inflammatory signs in the affected area. The main diagnostic hypotheses were fibrous hyperplasia and benign mesenchymal neoplasm.

The lesion was excised under local anesthesia. Microscopically, the lesion exhibited a well-circumscribed luminal papillary endophytic proliferation of squamous cells filling the lumen of an excretory duct of a minor salivary gland. The tumor was continuous with the superficial oral squamous epithelium (Fig. 1B), and it was arranged in islands of basaloid cells in a mild inflammatory fibrous stroma. Goblet cells, and microcysts were also found (Fig. 1C). There was no evidence of cytological atypia, although few mitotic Figures were observed (Fig. 1D).

**Figure 2:**
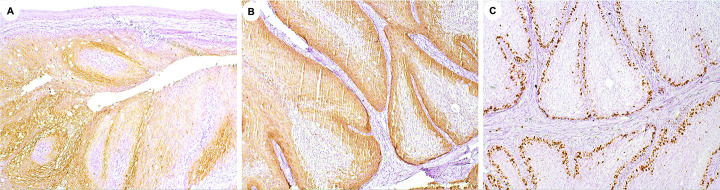
Immunohistochemical findings of the case. A: Diffuse positive staining with CK7, confirming the epithelial nature of most tumor cells (DAB, 100x). B: Cytoplasmatic expression of neoplastic cells for CK14 was also observed (DAB, 200x). C: Ki67 was expressed in about 20% of the tumor cells (DAB; 200x).

Immunohistochemical study was performed in 3 µm sections of formalin-fixed, paraffin-embedded tissues. The chromogen was diaminobenzidine tetrahydrochloride (DAB, Sigma-Aldrich, St Louis, MO, USA) and Carazzi hematoxylin was used for counterstaining. The tumor cells were diffusely immunoreactive for CK7 (Fig. 2A). CK14 was positive in some layers of the tumor islands (Fig. 2B), and Ki67 was positively restricted to the basal tumor layer (Fig. 2C). Based on these findings, the final diagnosis was OIDP. The patient has not presented any signs of recurrence so far.

## Discussion

Although the official description of OIDP was performed in 1982, a previous report of three indistinguishable lesions termed as papillary epidermoid adenoma had been published (10). Still, the tumor presents an uncertain incidence, and it is considered infrequent (1).

In accordance with our review ( Table 1), adults with a mean age of 51.9 years are mostly affected by OIDP. A slight male predominance was found. As we observed, the lip and buccal mucosa were the most involved sites (14 cases; 35%). Duration of OIDP is long, with a mean of 37.4 months. Clinically, the lesion manifested as an asymptomatic, submucous nodule, with or without ulceration (22 cases; 55%). The main diagnostic hypotheses reported were mucocele (8 cases; 18.2%), squamous cell papilloma (5 cases; 11.4%), and fibroma (3 cases; 6.8%).

As an attempt to elucidate the etiology of OIDP, some authors investigated the presence of human papillomavirus (HPV) and isolated the subtypes 6 and 11 (7,11). On the other hand, other studies failed to demonstrate this association (6,9,12,13). Sala-Pérez *et al*. (2013) (6) suggested that recurrent trauma may play an important role in the development of OIDP. The long duration of the lesion, and the involvement of areas usually exposed to trauma, such as the lower lip and cheek mucosa, may support such hypothesis. However, this hypothesis does not justify the involvement of areas less exposed to trauma, including upper lip and vestibular mucosa. Genetic alterations may represent a potential field of study for the understanding of OIDP etiopathogenesis [4,14], although most publications did not perform molecular analysis (Table 1).

Immunohistochemical analysis of OIDP is not contributory for tumor diagnosis (1). However, there it is likely that the cells of the excretory duct glands and cells of the mucous surface are associated in OIDP (9,12). Then, positivity for EMA and cytokeratins suggests that the excretory duct basal cells are responsible for the tumor origin (3,9,12,15). We observed that most publications did not perform immunohistochemistry, precluding a better comprehension of OIDP tumor origin and diagnosis (Table 1).

The histological differential diagnosis of OIDP include other salivary gland papillary tumors, such as sialoadenoma papilliferum, which exhibits exophytic growth, inflammatory connective tissue projections with acanthosis, and parakeratosis at the surface of the epithelium (4). Intraductal papilloma, in contrast to OIDP, forms a unicystic cavity (4,6). Another differential diagnosis is mucoepidermoid carcinoma, which exhibits cellular atypia and infiltrative growth pattern (1,5).

According to our findings, complete surgical removal remains the most appropriate option for OIDP treatment (10 cases; 100%). No recurrences (18 cases; 45%) have been reported after a mean follow-up time of 55 months. Complications of the lesion are related to incomplete removal (6).

In conclusion, OIDP is an uncommon neoplasm of minor salivary glands. Despite of this, it should be considered in the differential diagnosis of oral submucous nodules, especially those in the lower lip. Immunohistochemistry may be an important tool to determine the tumor origin and genetic analyses may be helpful to comprehend OIDP etiology.

## Figures and Tables

**Table 1:** Demographic and clinicopathological features of OIDP cases published in the literature.

Clinicopathological variables	n = 40	%
Sex	-	-
Female	18	45
Male	22	55
Age (mean age:51.9 yrs)	-	-
< 51.9 yrs	17	44.7
> 51.9 yrs	21	55.3
Site	-	-
Lip	14	35
Buccal mucosa	14	35
Floor of the mouth	3	7.5
Palate	6	15
Others^1^	3	7.5
Duration (mean:37.4 months)	-	-
< months	10	25
> months	2	5
ND	28	70
Symptoms	-	-
Pain	1	2.5
Asymptomatic	16	40
ND	23	57.5
Clinical presentation	-	-
Swelling	22	55
Ulcer	0	0
Swelling + ulcer	7	17.5
ND	11	27.5
Diagnostic hypothesis*	-	-
Mucocele	8	18.2
Papiloma	5	11.4
Fibroma	3	6.8
Lipoma	2	4.5
Benign salivary gland tumor	2	4.5
Malignant salivary gland tumor	1	2.3
Others²	4	9.1
ND	19	43.2
Immunohistochemistry	-	-
Yes	9	22.5
No	31	77.5
Molecular analysis	-	-
Yes	12	30
No	28	70
Treatment	-	-
Excision	40	100
Cryosurgery	0	0
ND	0	0
Follow-up time (mean: 55 months)	-	-
< 55 months	14	35
> 55 months	3	7.5
ND	23	57.5
Recurrence	-	-
Yes	0	0
No	18	45
ND	22	55

¹ Mandibular muco-buccal fold (1), oral mucosa NOS (1), mandibular vestibule (1). * For some cases, more than one hypothesis has been described. ² Condyloma acuminatum (1), soft tissue abscess (1), squamous cell carcinoma (1), benign tumor (1).

## Data Availability

The datasets used and/or analyzed during the current study are available from the corresponding author.
